# Dr. Harrington, on Digitalis

**Published:** 1800-03

**Authors:** Tho. Harrington

**Affiliations:** Piccadilly


### *To the Editors of the Medical and Phyſical Journal.*

GENTLEMEN,

IN your uſeful Journal ſo much has been ſaid (and moſtly very properly ſaid) relative to the *Digitalis*, that every liberal mind muſt feel a ſatisfaction in peruſing thoſe friendly efforts to aſcertain the real powers and utility of a medicine, thought by many to be of eminent ſervice in what has been deemed, in ſome ſtages, an incurable diſorder, the *pulmonary conſumption*. I muſt confeſs, the few trials I have made with the *Digitalis* incline me in its favour, and therefore, in your laſt Number, I was pleaſed with Dr. Kinglake's practice, in altering the common method in which patients uſually took their diet. The Doctor's method of recommending their receiving nutriment more frequent, and leſs in quantity, rationally appears to tend more to facilitate and leſs to clog the wheels of life; and every effort that will promote ſtrength in this diſorder is of much importance, and may greatly aſſiſt the operation of the *Digitalis*. I am now trying ſome experiments to aſcertain the qualities of this uſeful plant, and, ſhould they prove teſtimonies in its favour, a particular account of them ſhall be tranſmitted to you. In the mean time, I wiſh to notice the uſe of the *Digitalis* in ſome caſes of the *dropſy*, as mentioned by the late Dr. Darwin, and quoted by Dr. Ferriar in his recent publication on the *Digitalis*. But Dr. Ferriar having omitted to give in his work the extracts from the publication of his ingenious predeceſſor, and believing the treatiſe itſelf in but few hands, I am happy in preſenting them to your ſervice; and am,

GENTLEMEN,

With reſpect,

Your very humble ſervant,

*Piccadilly, Feb.* 10, 1800. THO. HARRINGTON.

---

It appears, that about the time (1778) when Mr. Darwin made the following extracts, the fox-glove was much in practice in Leiceſterſhire, and given with conſiderable ſucceſs in the dropſy. The annexed caſes are, therefore, related with deſign to aſcertain the particular kind of dropſy in which this plant appears preferable to other evacuents.

*Anasarca of the Lungs.*

1. A Lady, between forty and fifty years of age, had been indiſpoſed ſome time, was then ſeized with cough and fever, and and afterwards expectorated much digested mucus. This expectoration suddenly ceased, and a considerable difficulty of breathing supervened, with a pulse very irregular both in velocity and strength; she was much distressed at first lying down, and at first rising; but, after a minute or two, bore either of those attitudes with ease. She had no pain or numbness in her arms; she had no hectic fever, nor any cold shiverings; and the urine was in due quantity, and of the natural colour. The difficulty of breathing was twice considerably relieved by small doses of ipecacuhana, which operated upwards and downwards, but recurred in a few days; she was then directed a decoction of fox-glove (digitalis purpurea), prepared by boiling four ounces of the fresh leaves from two pints of water to one pint; to which were added two ounces of vinous spirit; she took three large spoonfuls of this mixture every two hours, till she had taken it four times; a continued sickness supervened, with frequent vomiting, and a copious flow of urine; these evacuations continued at intervals for two or three days, and relieved the difficulty of breathing. She had some relapses afterwards, which were again relieved by the repetition of the decoction of fox-glove.

2. A Gentleman, about sixty years of age, who had been addicted to an immoderate use of fermented liquors, and had been very corpulent, gradually lost his strength and flesh; had great difficulty of breathing, with legs somewhat swelled, and a very irregular pulse. He was very much distressed at first lying down, and at first rising from his bed, yet in a minute or two was easy in both those attitudes. He made straw coloured urine in due quantity, and had no pain or numbness of his arms. He took a large spoonful of the decoction of fox-glove, as above, every hour, for ten or twelve successive hours; had incessant sickness for about two days, and passed a large quantity of urine; upon which his breath became quite easy, and the swelling of his legs subsided; but, as his whole constitution was already sinking from the previous intemperance of his life, he did not survive more than three or four months.

### *Hydrops Pericardii.*

3. A Gentleman, of temperate life and sedulous application to business, between thirty and forty years of age, had long been subject, at intervals, to an irregular pulse; a few months ago he became weak, with difficulty of breathing, and dry cough. In this situation, a physician of eminence directed him to abstain from all animal food and fermented liquor; during which regimen all his complaints increased. He now became emaciated, and totally lost his appetite; his pulse very irregular both in velocity and strength, with great difficulty of breathing, and some

ſwelling of his legs; yet he could lie down horizontally in his bed, though he got little ſleep, and paſſed a due quantity of urine, and of the natural colour; no fullneſs or hardneſs could be perceived about the region of the liver; and he had no pain or numbneſs in his arm. One night he had a moſt profuſe ſweat all over his body and limbs, which quite deluged his bed, and for a day or two ſomewhat relieved his difficulty of breathing, and his pulſe became leſs irregular; this copious ſweat recurred three or four times, at the intervals of five or ſix days, and repeatedly alleviated his ſymptoms. He was directed one large ſpoonful of the above decoction of fox-glove every hour, till it procured ſome conſiderable evacuation; after he had taken it eleven ſucceſſive hours he had a few liquid ſtools, attended with a great flow of urine, which laſt had a dark tinge, as if mixed with a few drops of blood; he continued ſick, at intervals, for two days, but his breath became quite eaſy, and his pulſe quite regular; the ſwelling of his legs diſappeared, and his appetite and ſleep returned. He then took three grains of white vitriol twice a day, with ſome bitter medicines, and a grain of opium, with five grains of rhubarb, every night; was adviſed to eat fleſh meat, and ſpice, as his ſtomach would bear it, with ſmall beer, and a few glaſſes of wine; and had iſſues made in his thighs. He has ſuffered no relapſe.

4. A Lady, about fifty years of age, had, for ſome weeks, great difficulty of breathing, with very irregular pulſe, and conſiderable general debility; ſhe could lie down in bed, and the urine was in due quantity, and of the natural colour, and ſhe had no pain or numbneſs in her arms. She took one large ſpoonful of the above decoction of fox-glove every hour, for ten or twelve ſucceſſive hours; was ſick, and made a quantity of pale urine for about two days, and was quite relieved of the difficulty of breathing, and the irregularity of her pulſe. She then took a grain of opium, and five grains of rhubarb every night, for many weeks, with ſome ſlight chalybeate and bitter medicines; and has ſuffered no relapſe.

*Hydrops Thoracis,*

5. A Tradeſman, about fifty years of age, became weak and ſhort of breath, eſpecially on increaſe of motion, with pain in one arm, about the inſertion of the biceps muſcle. He obſerved he ſometimes in the night made an unuſual quantity of pale water. He took calomel, alum, and Peruvian bark, and all his ſymptoms increaſed; his legs began to ſwell conſiderably; his breath became more difficult, and he could not lie down in bed; but all this time he made a due quantity of ſtraw coloured water. The decoction of fox-glove was given, as in the preceding ceding cafes, which operated chiefly by purging, and feemed to relieve his breath for a day or two; but alfo feemed to contribute to weaken him. He became, after fome weeks, univerfally dropfical, and died comatofe.

6. A young lady of delicate conftitution, with light eyes and hair, and who had perhaps lived too abftemioufly, both in refpect to the quantity and quality of what fhe eat and drank, was feized with great difficulty of breathing, fo as to threaten immediate death. Her extremities were quite cold, and her breath felt cold to the back of one's hand. She had no fweats, nor could fhe lie down for a fingle moment; and had previoufly, and at prefent, complained of great weaknefs and pain, and numbnefs of both her arms; had no fwelling of her legs, nor thirft; water in due quantity and colour. Her fifter, about a year before, was afflicted with fimilar fymptoms, was repeatedly blooded, and died univerfally dropfical. A grain of opium was given immediately, and repeated every fix hours, with evident and amazing advantage; afterwards a blifter, with chalybeates, bitters, and effential oils were exhibited; but nothing had fuch eminent effect in relieving the difficulty of breathing, and coldnefs of her extremities, as opium, by the ufe of which, in a few weeks, fhe perfectly regained her health, and has, for more than two years, fuffered no relapfe.

### *Ascites.*

7. A young Lady, of delicate conftitution, having been expofed to great fear, cold, and fatigue, by the overturning of a chaife in the night, began with pain and tumour in the right hypochondrium; in a few months a fluctuation was felt throughout the whole abdomen; more diftinctly perceptible indeed about the region of the ftomach, fince the integuments of the lower part of the abdomen generally become thickened in this difeafe by a degree of anafarca. Her legs were not fwelled; no thirft; water in due quantity and colour. She took the foxglove fo as to induce ficknefs and ftools, but without abating the fwelling, and was obliged at length to fubmit to the operation of tapping.

8. A Man, about fixty-feven, who had long been accuftomed to fpirituous potations, had fome time laboured under afcites; his legs fomewhat fwelled; his breath eafy in all attitudes; no appetite; great thirft; urine in exceedingly fmall quantity, very deep coloured and turbid; pulfe quick. He took the foxglove in fuch quantity as vomited him, and induced ficknefs for two days; but procured no flow of urine, or diminution of his fwelling; but was thought to leave him confiderably weaker.

9. A

9. A corpulent man, accuſtomed to large potations of fermented liquors, had vehement cough, difficult breathing, anaſarca of his legs, thighs, and hands, and conſiderable tumour, with evident fluctuation of his abdomen; his pulſe was equal, his urine in ſmall quantity, of deep colour, and turbid. Theſe ſwellings had been twice conſiderably abated by draſtics cathartics. He took three ounces of a decoction of fox-glove, made by boiling one ounce of the freſh leaves in a pint of water, every three hours, for two whole days; it then began to vomit and purge him violently, and promoted a great flow of urine; he was, by theſe evacuations, compleatly emptied in twelve hours. After two or three months all theſe ſymptoms returned, and were again relieved by the uſe of the fox-glove; and thus, in the ſpace of three years, he was about ten times evacuated, and continued all that time his uſual potations; excepting at firſt the medicine operated only by urine, and did not appear conſiderably to weaken him. The laſt time he took it, it had no effect; and a few weeks afterwards he vomited a great quantity of blood, and expired.

QUERE. "Might not the fox-glove be ſerviceable in hydrocephalus internus, in hydrocele, and in white ſwellings of the joints?"——It certainly requires judgment and great caution in its uſe. Some authenticated inſtances of its fatal effects will be ſhortly ſent to the Editors.

